# Correlation between family physician’s direct advice and pneumococcal vaccination intention and behavior among the elderly in Japan: a cross-sectional study

**DOI:** 10.1186/s12875-018-0841-3

**Published:** 2018-09-05

**Authors:** Mariko Higuchi, Keiichiro Narumoto, Takahiro Goto, Machiko Inoue

**Affiliations:** 1 The Department of Internal Medicine, Tsukuba Central Hospital, 1589-3 Kashiwadachou, Ushiku, Ibaraki 300-1211 Japan; 2 The Department of Obstetrics and Gynecology, Kikugawa Municipal General Hospital, 1632 Higashiyokoji, Kikugawa, Shizuoka 439-0022 Japan; 3 Shizuoka Family Medicine Residency Program, 1632 Higashiyokoji, Kikugawa, Shizuoka 439-0022 Japan; 4 Yu Pharmacy, 15-1 Otsudori, Shimada, Shizuoka 427-0056 Japan; 5 The Department of Family and Community Medicine, Hamamatsu University School of Medicine, 1-20-1 Handayama, Higashi-ku, Hamamatsu, Shizuoka 431-3192 Japan

**Keywords:** Family practice, Preventive medicine, 23-valent pneumococcal polysaccharide vaccine, Physician’s advice

## Abstract

**Background:**

Vaccination is an important element of health maintenance in family medicine. The 23-valent pneumococcal polysaccharide vaccine (PPSV23) is highly recommended for the elderly, but its uptake is low in Japan. Primary care system remains under development and preventive services tend to be neglected in the Japanese medical practice. The study aims to investigate the association between family physician’s recommendations for PPSV23 during outpatient care and PPSV23 vaccination intention and behavior in the elderly.

**Method:**

We conducted a cross-sectional study with a questionnaire at a family medicine clinic in a rural area in Japan. The participants were over the age of 65 without dementia who had maintained a continuity with the clinic. The questionnaire inquired PPSV23 vaccination status, family physician’s advice for PPSV23, socio-demographics, and the constructs in the Health Belief Model. We defined those who had had vaccination intention and behavior as “PPSV23 vaccinated group” and those who had no vaccination and uncertainty about being or no intention to be vaccinated in the future as “PPSV23 unvaccinated group.” We used chi-square test for correlation between physician’s advice and PPSV23 vaccination/intention, univariate and multivariate logistic regression analysis for factors related to the vaccination/intention, and descriptive analysis for reasons for reluctance to the vaccination.

**Results:**

We analyzed 209 valid responses. There were 142 participants in the PPSV23 vaccinated group and 67 in the PPSV23 unvaccinated group. The PPSV23 vaccination group was more likely to have had their physician’s advice (80.2% vs 21.3%, *p* < 0.001). Multivariate logistic regression analysis showed a significant association between PPSV23 vaccination and their physician’s recommendation (OR 8.50, 95%CI 2.8–26.0), awareness of PPSV23 (OR 8.52, 95%CI 2.1–35.0), and the perceived effectiveness of PPSV23 (OR 4.10, 95%CI 1.2–13.9). The reasons for reluctance to get vaccinated included lack of understanding of PPSV23, lack of physician’s recommendations, and concerns about side effects of PPSV23.

**Conclusion:**

Family physician’s recommendation was positively correlated with PPSV23 vaccination intention and behavior in the elderly. This reinforces the importance of providing preventive services during time-constrained outpatient care, even in medical systems where it is undervalued.

**Electronic supplementary material:**

The online version of this article (10.1186/s12875-018-0841-3) contains supplementary material, which is available to authorized users.

## Background

Preventive care is an important element in family medicine. However, it tends to be undervalued, as curative medicine is mainstream in Japanese medical practice [1]. Some Japanese primary care physicians experience difficulties practicing preventive medicine in their busy outpatient care because of restricted hours of consultation, perceived difficulties encouraging behavior changes in patients, and the lack of seeing the effectiveness of preventive medicine [2]. In addition, under the current medical billing system, providing preventive services are not adequately paid. Hospital-employed physicians typically need to handle outpatient care in parallel with inpatient management. Therefore, outpatient practice becomes a large burden as they already spend a significant amount of time working on a variety of tasks for inpatient care [3]. These factors restrict the practice of preventive medicine in Japan.

Vaccination is one of the most important parts of health maintenance. The 23-valent pneumococcal polysaccharide vaccine (PPSV23) is recommended for the elderly since it lowers the risks of invasive pneumococcal disease [4] and pneumococcal pneumonia. [5] Pneumonia holds 3rd place for cause of death in the elderly [6], and streptococcus pneumoniae is the most dominant causative bacteria, accounting for 17 to 28% in cases of pneumonia in adults [7–9]. In Japan, the PPSV23 has proven to be effective in reducing the incidence of pneumonia in general as well as pneumococcal pneumonia specifically [10]. However, receiving the PPSV23 vaccine was voluntary until October 1, 2014, when the vaccine was added to the list of routine vaccinations, therefore the uptake of the PPSV23 vaccination was extremely low at just 17.5% in March, 2012 [11]. While the Centers for Disease Control and Prevention recommend both 13-valent pneumococcal conjugate vaccine (PCV13) and PPSV23 for the elderly over the age of 65 [12], only PPSV23 is currently part of routine vaccinations with public health subsidies available every 5 years after 65 years of age in Japan.

Several facilitators of recommended vaccination have been reported: recommendations from family members or friends [13–15], notifications sent to individuals and homepages for public relations purposes set up by each local municipality [16], having knowledge about the vaccine [13, 17], personal record of vaccination [15], and the perceived severity of diseases such as pneumonia, as well as the safety and perceived effectiveness of the vaccine [15, 17, 18]. On the other hand, barriers against vaccination in existing studies included: lack of knowledge about the vaccine, anxiety about its side effects [13, 19, 20], doubting the effectiveness of the vaccine, lack of interest, the cost of self-pay, as well as difficulties making hospital visits due to lack of time [20].

Because preventive medicine provided with the concept of continuity is important in primary care, family physicians have a crucial role in ensuring health maintenance of their patients. Maintaining a continuous relationship with the same family physician or medical institution was positively correlated with receipt of preventive services [21]. In the previous study in Britain, the advice given by general practitioners had the most significant influence on patient’s decisions to get PPSV23 [22].

However, in Japan, there is no research on the influence of advice by family physicians on patient’s receiving preventive services, specifically PPSV 23 vaccination. Additionally, no investigation into the other factors affecting PPSV23 vaccination in the elderly exists in the Japanese primary care setting.

The purpose of this study was to investigate (1) the association between family physician’s direct advice and PPSV23 vaccination intention and behavior in the elderly, (2) factors related to their PPSV23 vaccination intention and behavior, and (3) reasons for reluctance to receive PPSV23 vaccination.

## Methods

This is a cross-sectional study of the elderly who regularly visit a single outpatient healthcare clinic, Kikugawa Family Medicine Center, in Shizuoka prefecture, Japan. The study was approved by the Ethics Committee of Kikugawa General Hospital and permission and approval to the investigation were obtained from the clinic.

### Setting

The study was conducted at a family medicine clinic in a rural community with a population of approximately 50,000 people. At the clinic, two certified family physicians and 4 family medicine residents were running a group practice at the time of the investigation. The residents had been educated in preventive care as an important element of family medicine by a certified family physician faculty. Therefore, all the physicians at the clinic shared the common ground of a value of preventive medicine in primary care. However, their actual practice might vary according to multiple factors including outpatient time constraint, the complexity of patient health issues and the extent of establishment of patient-physician relationship. Out of the total of 18,756 patients who came to the clinic in 2013, 9094 (48.5%) were elderly patients over the age of 65. The period between clinic visits, depending on their medical conditions and needs, averaged anywhere from 1 week to 3 months. With national health insurance coverage and patients having free access to almost any kind of medical institution, the average outpatient consultation time per patient is between 3 and 15 min in the clinic, according to the complexity of the health problems of each patient.

### Participants

We used the convenience sampling method, approaching and distributing the questionnaire to the patients who came to the clinic from November 1, 2013 until a total 500 responses were collected based on predetermined sampling calculation as mentioned in analysis. Inclusion criteria for those to whom the questionnaire was distributed were (1) over the age of 65 as of September 1, 2013, (2) capable of giving their consent to participate in the study and (3) agreed to the study participation. Written informed consent was obtained from all participants prior to the study. After data collection, we then reviewed their electronic medical record (EMR) to confirm the additional inclusion criteria: (4) having had at least 3 consultations in the clinic from August 1, 2011, when the clinic opened, to September 1, 2013 and (5) having no diagnosis of dementia.

### Data collection

Participants received the paper-based questionnaire and were asked to complete it while waiting for their outpatient consultation. With visual impairments or difficulty in interpreting the questions, they could have assistance from family members or the research staff at the clinic.

### Data confirmation by EMR

After collecting all responses to the questionnaire, we checked the number of clinic visits from the aforementioned period of time (August, 2011 to September, 2013) to evaluate continuity to the clinic as one of the inclusion criteria. We also reviewed the vaccination records of PPSV23 in EMR to validate the answer of the vaccination status in the questionnaire and any documentation regarding the diagnosis of dementia. We defined diagnosis of dementia if one of the following criteria were met: (1) dementia had been registered on the list of diagnosis, (2) there had been a record of Mini-Mental State Examination (MMSE), scoring less than 24 points, or (3) dementia was on the problem list in the progress note.

We excluded the data if the participants did not meet inclusion criteria. We also excluded if there was discordance between responses about vaccination status in the questionnaire and vaccination records in EMR.

### The questionnaire

The questionnaire was comprised of a total of 22 items.

First, the questionnaire asked the participants (1) whether they were aware of PPSV23, (2) whether their family physicians had recommended PPSV23 vaccination for their age, and (3) their PPSV23 vaccination status. For those who had not been vaccinated, the questionnaire asked them to choose one from the following three options about their future vaccination intentions (Additional file 1): “I plan to get vaccinated in the future (future vaccination intention).”, “I have decided not to get vaccinated (no intention to future vaccination).”, and “I don’t know if I will get vaccinated or not (uncertainty about future vaccination).” As for those who had not been vaccinated for PPSV23 and who also showed uncertainty about or no intention to be vaccinated in the future, they were asked to choose a single or multiple reasons for their reluctance from 12 items [14, 18–20].

Secondly, the questionnaire asked for the participant’s socio-demographic information, including age, gender, educational background, personal financial situation, smoking habits, their medical history of pneumonia and other respiratory diseases, their family history of pneumonia, awareness of PPSV23 and vaccine-related public subsidies, perceived current health conditions, and constructs used in Health Belief Model (HBM) [23–25], by reference to the factors that influenced people’s intention to receive vaccination in the previous studies. The HBM is a conceptual model that is widely used to analyze health behaviors. In line with the context of the study, the participants were asked questions about (1) perceived susceptibility to the common cold, (2) perceived susceptibility to pneumonia, (3) perceived severity of pneumonia, (4) perceived effectiveness of PPSV23, and (5) a sense of economic burden as perceived barriers to receiving PPSV23, using a 5-point Likert scale.

### Analysis

We defined the respondents who had a history of PPSV23 vaccination confirmed in EMR or who had not been vaccinated but showed the future vaccination intention (“I plan to get vaccinated in the future.”), as the “PPSV23 vaccinated group.” On the other hand, we defined the respondents who had no history of PPSV23 vaccination and who showed uncertainty about or no intention to future vaccination (“I have decided not to get vaccinated.” or “I don’t know if I will get vaccinated or not.”) as the “PPSV23 unvaccinated group”.

It was calculated that a total of 174 responses were necessary in this study (significant level = 0.05, power = 80%, effect size = 0.3) [26, 27]. Since the participants were elderly people over the age of 65, we assumed the response rate or valid response would be low. Therefore, we decided to distribute the questionnaire to 500 people.

We used chi-square test to analyze the association between family physician’s advice and PPSV23 vaccination intention/behavior. We applied univariate logistic regression analysis to examine the factors related to PPSV23 vaccination intention/behavior. For multivariate logistic regression analysis, all factors in univariate analysis associated with PPSV23 vaccination at *p* < 0.2 were included.

The dependent variable was PPSV23 vaccination intention/behavior. Independent variables included family physician’s direct advice, participant’s demographics (age, gender, educational background, personal financial situation, smoking habits, past medical and family history of pneumonia, awareness of PPSV23 and vaccine-related public subsidies, perceived current health conditions), and constructs adapted from Health Belief Model (HBM). We analyzed the responses on the 5-point Likert scale by separating them into the top two and the lower 3 answers as nominal variables. We conducted descriptive analysis on the PPSV23 unvaccinated group’s reasoning for reluctance to be vaccinated. The analysis was carried out with R version 3.2.0. statistical software [28].

## Results

**Fig. 1:**
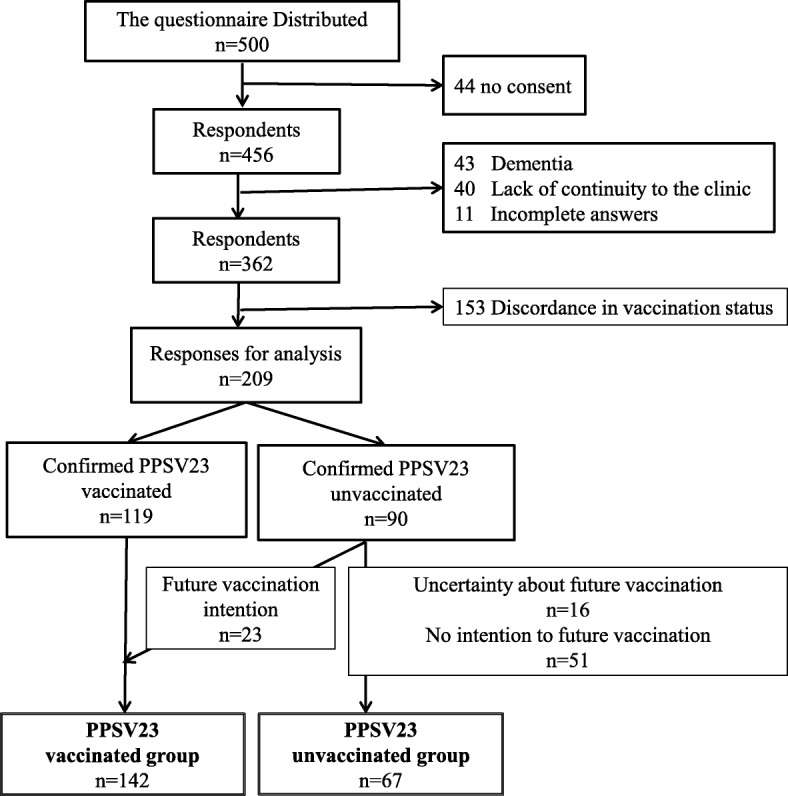
Selection of participants and definitions of PPSV23 vaccinated and unvaccinated group. It shows the process of selection of the participants and depicts the definitions of PPSV23 vaccinated and unvaccinated group

In the study, “PPSV23 vaccinated group” included those who had had PPSV23 vaccination intention/behavior at the time of the investigation. Of 500 elder patients to whom we distributed the questionnaires, 456 patients agreed to participate in the study. Of 456 respondents, we excluded 247 responses (40: lack of continuity, 43: dementia, 153: discordance in vaccination status in between the questionnaire and EMR, 11: incomplete responses), resulting in a total of 209 valid responses for analysis (Fig. 1).

A hundred nineteen respondents were confirmed vaccinated with PPSV23, and 90 respondents were not vaccinated. Among the unvaccinated, 23 respondents had future vaccination intention, therefore by definition, there were 142 respondents in PPSV23 vaccinated group and 67 in PPSV23 unvaccinated group.

Table 1 shows characteristics of the participants including 153 respondents whose reports of PPSV23 vaccination status were discordant in between the questionnaire and EMR, categorized as “uncertain PPSV23 vaccine status group.” The average ages of PPSV23 vaccinated, unvaccinated and uncertain vaccine status group were 77.6, 72.7, 74.2, respectively. The PPSV23 vaccinated group were more likely to report poor subjective health (72.6% vs. 63.5%, 64.9%), subjectively rated their financial situations as comfortable (12.9% vs 3.2%, 6.5%), and perceived effectiveness of PPSV23 (75.2% vs. 33.3%, 56.6%). The respondents in the PPSV23 vaccinated group were more likely to report family physician’s advice on PPSV23 vaccination (78.9% vs 20.3%, 58.5%). Univariate logistic regression analysis revealed that PPSV23 vaccination intention/behavior was significantly correlated with physician’s advice (*p* < 0.001), age (*p* < 0.001), awareness of PPSV23 (*p* < 0.001) and vaccination subsidies (*p* < 0.001), perceived susceptibility to the common cold (*p* = 0.016), perceived seriousness of pneumonia (*p* = 0.027), and perceived the effectiveness of PPSV23 (*p* < 0.001) [Table 2].

**Table 1:** Characteristics of study participants

		PPSV23 vaccinated group (*n* = 142) n (%) or Mean	PPSV23 unvaccinated group (*n* = 67) n (%) or Mean	Uncertain PPSV23 vaccine status group (*n* = 153) n (%) or Mean
Age (±S.E.)		77.6 ± 0.6	72.7 ± 1.0	74.2 ± 0.9
Sex	Male	64 (45.1)	30 (44.8)	71 (46.4)
	Female	78 (54.9)	37 (55.2)	82 (53.6)
Personal medical history of pneumonia	Yes	5 (4.1)	6 (9.4)	8 (10.4)
	No	118 (95.9)	58 (90.6)	69 (89.6)
Personal medical history of respiratory disease	Yes	15 (12.6)	5 (7.8)	4 (5.0)
	No	104 (87.4)	59 (92.2)	76 (95)
Family history of pneumonia	Yes	10 (7.9)	8 (12.7)	10 (11.5)
	No	116 (92.1)	55 (87.3)	77 (88.5)
Regular health check-ups^*^	Yes	112 (95.7)	59 (98.3)	70 (87.5)
	No	5 (4.3)	1 (1.7)	10 (12.5)
Current smoking habits	Smoker	14 (10.4)	4 (6.3)	7 (7.4)
	Non-smoker	120 (89.6)	60 (93.7)	87 (92.6)
Subjective state of health	Good	37 (27.4)	23 (36.5)	33 (35.1)
	Not good	98 (72.6)	40 (63.5)	61 (64.9)
Subjective sense of economic conditions	Comfortable	17 (12.9)	2 (3.2)	6 (6.5)
	Struggling	115 (87.1)	60 (96.8)	86 (93.5)
Highest level of education completed	Elementary or junior high school	67 (50.4)	32 (51.6)	45 (48.9)
	High school or higher	66 (49.6)	30 (48.4)	47 (51.1)
Living alone or with others	With others	111 (92.5)	54 (87.1)	68 (94.4)
	Alone	9 (7.5)	8 (12.9)	4 (5.6)
Necessity for transportation for clinic visits	Necessary	42 (31.1)	14 (21.9)	31 (33.7)
	Unnecessary	93 (68.9)	50 (78.1)	61 (66.3)
Perceived susceptibility to common colds ^**^	Yes	43 (33.6)	10 (16.4)	27 (31.0)
	No	85 (66.4)	51 (83.6)	69 (69.0)
Perceived susceptibility to pneumonia^**^	Yes	17 (13.2)	3 (4.8)	18 (20.5)
	No	112 (86.8)	59 (95.2)	70 (79.5)
Perceived severity of pneumonia^**^	Yes	42 (34.4)	12 (18.8)	34 (40.0)
	No	80 (65.6)	52 (81.2)	51 (60.0)
Perceived effectiveness of PPSV23 ^**^	Yes	91 (75.2)	21 (33.3)	47 (56.6)
	No	30 (24.8)	42 (66.7)	36 (43.4)
Perceived barriers to PPSV23 (sense of economic burden)^**^	Yes	44 (35.5)	18 (30.5)	27 (35.1)
	No	80 (64.5)	41 (69.5)	50 (64.9)
physician’s recommendation	Yes	105 (78.9)	13 (20.3)	38 (58.5)
	No	28 (21.1)	51 (79.7)	27 (41.5)
Awareness of PPSV23	Yes	120 (92.3)	28 (41.8)	69 (76.7)
	No	10 (7.7)	39 (58.2)	21 (23.3)
Awareness of public subsidies	Yes	37 (42.0)	8 (14.3)	18 (34.0)
	No	51 (48.0)	48 (85.7)	35 (66.0)
Difficulty completing the questionnaire	Completed with help.	30 (22.7)	13 (20.6)	13 (14.8)
	Completed without help.	102 (77.3)	50 (79.4)	75 (85.2)

*:Regardless of the frequency of health check-ups, if patients had regular medical check-ups within the last 5 years, the answers were put into the “Yes” category

**:The responses on 5-point Likert scale were separated into the top 2 group and the lower 3 group

The table shows demographic background of the participants and their responses for Health Belief Model related questions. Uncertain PPSV23 vaccine status group was defined by the participants whose reports of PPSV23 vaccination status in the questionnaire and PPSV23 vaccination records in electronic medical record were not consistent

**Table 2:** Univariate logistic regression analysis for factors related to PPSV23 vaccination intention and behavior

	OR	95%CI	*p*-value
Age	1.09	1.05–1.14	<0.001
Sex	1.01	0.56–1.81	0.968
Personal medical history of pneumonia	0.41	0.12–1.40	0.150
Personal medical history of respiratory disease	1.70	0.59–4.92	0.326
Family history of pneumonia	0.59	0.22–1.58	0.297
Regular health check-ups	0.38	0.04–3.33	0.381
Current smoking habits	1.75	0.55–5.55	0.341
Subjective state of health	0.66	0.35–1.24	0.196
Subjective sense of economic conditions	4.43	0.99–19.84	0.051
Highest level of education completed	0.95	0.52–1.74	0.872
Living alone or with others	1.83	0.67–5.00	0.240
Necessity of transportation to the clinic	1.61	0.80–3.23	0.178
Perceived susceptibility to common colds	2.58	1.19–5.58	0.016
Perceived susceptibility to pneumonia	2.99	0.84–10.60	0.090
Perceived severity of pneumonia	2.27	1.10–4.72	0.027
Perceived effectiveness of PPSV23	6.07	3.11–11.82	<0.001
Perceived barriers to PPSV23 (sense of economic burden)	1.25	0.64–2.44	0.507
Physician’s recommendation	14.70	7.03–30.77	<0.001
Awareness of PPSV23	16.70	7.45–37.47	<0.001
Awareness of public subsidies	4.35	1.84–10.28	<0.001
Difficulty in completing the questionnaire	1.13	0.54–2.34	0.741

There were significant relationships between PPSV23 vaccination and the following factors: physician’s recommendations, awareness of PPSV23 and public vaccination subsidies, age, perceived susceptibility to common colds, perceived severity of pneumonia, and perceived effectiveness of PPSV23 (*p* < 0.05)

Multivariate logistic regression analysis showed physician’s advice was significantly associated with PPSV23 vaccination (OR 8.50, 95%CI 2.8–26.0). The other factors that were positively correlated with vaccination were awareness of PPSV23 (OR 8.52, 95%CI 2.1–35.0) and perceived effectiveness of PPSV23 (OR 4.10, 95%CI 1.2–13.9) [Table 3].

**Table 3:** Multivariate logistic regression analysis for factors^*^ related to PPSV23 vaccination intention and behavior

	OR	95%CI	*p*-value
Age	1.05	0.96–1.16	0.269
Personal medical history of pneumonia	0.12	0.01–1.31	0.083
Subjective state of health	0.69	0.20–2.36	0.554
Subjective sense of economic conditions	3.76	0.30–47.84	0.307
The necessity for transportation to the clinic	1.04	0.23–4.67	0.961
Perceived susceptibility to colds	1.17	0.26–5.33	0.836
Perceived susceptibility to pneumonia	7.02	0.50–98.71	0.149
Perceived severity of pneumonia	1.11	0.22–5.53	0.903
Perceived effectiveness of PPSV23	4.10	1.21–13.86	0.023
Physician’s recommendations	8.50	2.78–25.99	<0.001
Awareness of PPSV23	8.52	2.07–35.03	0.003
Awareness of public subsidies	1.82	0.51–6.52	0.356

^*^: The independent variables which were associated with PPSV23 at *p* < 0.20 in univariate logistic regression analysis were incorporated to multivariate logistic regression analysis

There was a significant relationship between the PPSV23 vaccine status and physician’s direct advice, awareness of PPSV23, and the perceived effectiveness of PPSV23.

**Fig. 2:**
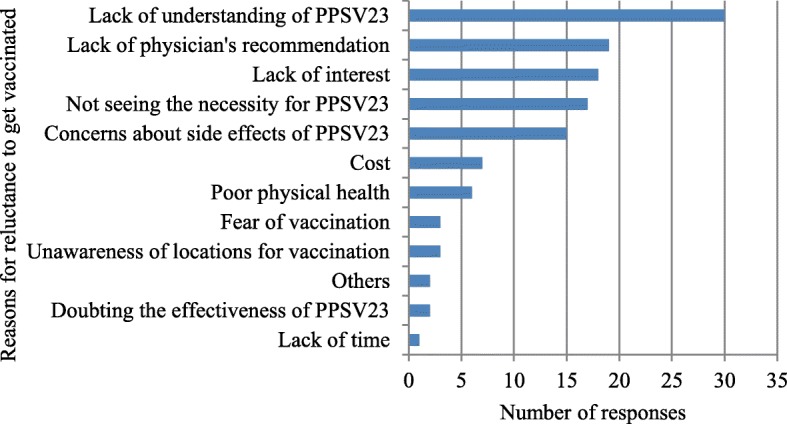
Reasons for reluctance to get vaccinated in PPSV23 unvaccinated group. It shows the number of responses for each reason for reluctance to get vaccinated in the questionnaire with multiple answers allowed. The question was asked only to the PPSV23 unvaccinated group (*n* = 67)

Reasons for not getting vaccinated with PPSV23 included “lack of understanding of PPSV23”, “lack of recommendations from their doctors”, “lack of interest in PPSV23”, “lack of perceived value of the vaccine”, and “concerns about the side effects of PPSV23” [Fig. 2].

We conducted sensitivity analysis using different definitions of PPSV23 vaccinated or unvaccinated group. The results from the sensitivity analysis did not differ significantly compared with those from the analysis using the original definition of outcome. First, we carried out an analysis for “confirmed PPSV23 vaccinated (n=119)” and “confirmed PPSV23 unvaccinated (n=90)” groups without intention to get vaccinated taken into consideration for definition of dependent variable. Physician advice was significantly correlated with PPSV23 vaccination in multivariate regression analysis (OR 182, 95%CI 19–1757). Second, since a substantial number of the participants were excluded due to discordance in vaccine status in between the questionnaire and EMR, we analyzed the data based on the definition of PPSV23 vaccine status by either vaccine records in EMR or reports in the questionnaire alone. When the former definition used, a total 362 participants were included in the analysis, where there were 225 (62.2%) and 137 participants in PPSV23 vaccinated and unvaccinated group, respectively. Physician’s recommendation remained positively correlated with PPSV23 vaccination (OR 4.73, 95%CI 2.33–9.62) [Tables 4, 5]. When the latter definition used, a total 255 responses in the questionnaire were valid and there were 163 (63.9%) and 92 participants in PPSV23 vaccinated and unvaccinated group, respectively. Physician’s recommendation was still positively correlated with PPSV23 vaccination (OR 7.37, 95%CI 2.83–19.22) [Tables 6, 7].

**Table 4:** Univariate logistic regression analysis for factors related to PPSV23 vaccination intention and behavior (PPSV23 vaccination status defined by electronic medical record)

	OR	95%CI	*p*-value
Age	1.01	0.98–1.12	0.234
Sex	1.03	0.57–1.85	0.953
Personal medical history of pneumonia	0.78	0.30–2.02	0.615
Personal medical history of respiratory disease	0.87	0.42–1.79	0.695
Family history of pneumonia	0.46	0.21–1.01	0.053
Regular health check-ups	0.80	0.27–2.39	0.696
Current smoking habits	1.42	0.57–3.52	0.449
Subjective state of health	0.72	0.43–1.20	0.203
Subjective sense of economic conditions	3.11	1.04–9.31	0.043
Highest level of education completed	1.14	0.70–1.86	0.590
Living alone or with others	1.09	0.43–2.74	0.852
Necessity of transportation to the clinic	1.50	0.87–2.59	0.142
Perceived susceptibility to common colds	2.56	1.39–4.69	0.002
Perceived susceptibility to pneumonia	2.73	1.16–6.46	0.021
Perceived severity of pneumonia	1.79	1.03–3.10	0.038
Perceived effectiveness of PPSV23	3.11	1.86–5.22	<0.001
Perceived barriers to PPSV23 (sense of economic burden)	1.27	0.74–2.18	0.388
Physician’s recommendation	7.52	4.28–13.23	<0.001
Awareness of PPSV23	4.90	2.76–8.72	<0.001
Awareness of public subsidies	1.47	0.79–2.73	0.227
Difficulty in completing the questionnaire	1.44	0.76–2.73	0.263

The PPSV23 vaccinated and unvaccinated groups were defined by the vaccination records in electronic medical record only, irrespective of the reports of vaccination status in the questionnaire. There were significant relationships between PPSV23 vaccination and the following factors: subjective sense of economic conditions, perceived susceptibility to common colds, perceived susceptibility to pneumonia, perceived severity of pneumonia, perceived effectiveness of PPSV23, physician’s direct advice and awareness of PPSV23 (*p* < 0.05)

**Table 5:** Multivariate logistic regression analysis for factors^*^ related to PPSV23 vaccination intention and behavior (PPSV23 vaccination status defined by electronic medical record)

	OR	95%CI	*p*-value
Family history of pneumonia	0.35	0.12–1.07	0.066
Subjective sense of economic conditions	3.04	0.76–12.26	0.117
The necessity for transportation to the clinic	1.34	0.61–2.95	0.470
Perceived susceptibility to common colds	2.03	0.81–5.08	0.132
Perceived susceptibility to pneumonia	1.29	0.35–4.77	0.699
Perceived severity of pneumonia	0.96	0.39–2.34	0.924
Perceived effectiveness of PPSV23	2.09	0.98–4.47	0.056
Physician’s recommendations	4.73	2.33–9.62	<0.001
Awareness of PPSV23	2.87	1.18–6.96	0.020

^*^: The independent variables which were associated with PPSV23 at *p* < 0.20 in univariate logistic regression analysis were incorporated to multivariate logistic regression analysis

The PPSV23 vaccinated and unvaccinated groups were defined by the vaccination records in electronic medical record only, irrespective of the questionnaire responses. There were significant relationships between PPSV23 vaccination and physician’s direct advice (*p* < 0.001) and awareness of PPSV23 (*p* = 0.02)

**Table 6:** Univariate logistic regression analysis for factors related to PPSV23 vaccination intention and behavior (PPSV23 vaccination status defined by questionnaire)

	OR	95%CI	*p*-value
Age	1.05	0.96–1.17	0.312
Sex	1.07	0.63–2.01	0.972
Personal medical history of pneumonia	0.33	0.12–0.95	0.039
Personal medical history of respiratory disease	1.21	0.56–2.61	0.634
Family history of pneumonia	1.06	0.44–2.51	0.902
Regular health check-ups	1.14	0.35–3.73	0.823
Current smoking habits	1.29	0.51–3.30	0.592
Subjective state of health	0.71	0.41–1.25	0.235
Subjective sense of economic conditions	2.82	0.92–8.62	0.069
Highest level of education completed	0.98	0.58–1.67	0.949
Living alone or with others	1.88	0.73–4.84	0.190
Necessity of transportation to the clinic	1.45	0.80–2.64	0.224
Perceived susceptibility to common colds	1.67	0.90–3.09	0.105
Perceived susceptibility to pneumonia	1.38	0.60–3.17	0.447
Perceived severity of pneumonia	1.47	0.82–2.66	0.197
Perceived effectiveness of PPSV23	4.46	2.49–7.99	<0.001
Perceived barriers to PPSV23 (sense of economic burden)	1.03	0.57–1.83	0.929
Physician’s recommendation	7.82	4.29–14.27	<0.001
Awareness of PPSV23	14.32	6.67–30.75	<0.001
Awareness of public subsidies	4.81	2.27–10.16	<0.001
Difficulty in completing the questionnaire	1.11	0.57–2.15	0.756

The PPSV23 vaccinated and unvaccinated groups were defined by the questionnaire responses only, irrespective of the vaccination records in electronic medical record. There were significant positive relationships between PPSV23 vaccination and the following factors: perceived effectiveness of PPSV23, physician’s recommendations, and awareness of PPSV23 and public subsidies, while an inverse relationship was observed between PPSV23 vaccination and personal medical history of pneumonia (*p* < 0.05)

**Table 7:** Multivariate logistic regression analysis for factors^*^ related to PPSV23 vaccination intention and behavior (PPSV23 vaccination was defined by questionnaire responses only)

	OR	95%CI	*p*-value
Personal medical history of pneumonia	0.27	0.04–1.81	0.179
Subjective sense of economic conditions	3.63	0.38–34.95	0.265
Living alone or with others	1.60	0.28–9.02	0.595
Perceived susceptibility to colds	0.73	0.24–2.23	0.583
Perceived severity of pneumonia	1.24	0.35–4.42	0.744
Perceived effectiveness of PPSV23	2.90	1.03–8.15	0.044
Physician’s recommendations	7.37	2.83–19.22	<0.001
Awareness of PPSV23	8.74	2.23–34.31	<0.001
Awareness of public subsidies	1.97	0.68–5.71	0.214

^*^: The independent variables which were associated with PPSV23 at *p* < 0.20 in univariate logistic regression analysis were incorporated to multivariate logistic regression analysis

The PPSV23 vaccinated and unvaccinated groups were defined by the questionnaire responses only, irrespective of the vaccination records in electronic medical record. There was a significant relationship between PPSV23 vaccination and perceived effectiveness of PPSV23 (*p* < 0.05), physician’s direct advice (*p* < 0.001) and awareness of PPSV23 (*p* < 0.05)

## Discussion

The study revealed family physician’s direct advice was significantly correlated with PPSV23 vaccination intention/behavior in the elderly in the Japanese primary care setting. To our knowledge, this is the first study in Japan to examine the value of preventive service provided by family physicians during the busy outpatient continuity care.

Among the principles in family medicine, using illness visits as opportunities for preventive medicine [29] is important to achieve comprehensive care. However, the focus in outpatient encounters in primary care is typically more on laboratory tests and treatments with prescriptions rather than providing preventive services due to medical service fees [1, 2]. Therefore, it is questionable whether or not many healthcare providers place a value on preventive medicine during busy outpatient visits. When examining geriatric patients, primary care physicians need to manage not only polypharmacy and multimorbidity but also newly developed acute health issues within constricted outpatient time. Furthermore, it is not uncommon for them to need to address any concern from the patient’s caretakers. It is possible that those factors make it challenging for family physicians to practice preventive medicine. Given the fact that frequency of visiting the clinic is relatively high at 1–2 months intervals in Japan, family physicians might be able to continue to provide preventive services little by little in each visit. However, it has been pointed out that the number of consultations is not necessarily proportional to the providing of preventive healthcare [30]. Under such circumstances, the study results reinforced an important role of family physicians in preventive medicine even during a busy outpatient encounter in the Japanese medical system.

Continuity of care is one of the most important disciplines of family medicine, and family physicians build cumulative knowledge of their patients in the process [31]. It has been suggested that updating immunization records is related to “preference for usual physicians” or “physicians’ accumulated knowledge of patients” [32]. In this study, we did not use specific instruments to measure continuity [33]. However, we took a “chronological” continuity into account [34], and it is one of the strengths of our study that, in that context, family physician’s advice was associated with the recommended vaccination intention/behavior for the elderly.

“Perceived effectiveness of PPSV23” as facilitators of vaccination and “lack of interest in PPSV23” or “concerns about the side effects of PPSV23” as the reasons for reluctance to get vaccinated were regarded as patient’s perceptions of the vaccine, which could indicate the importance of providing preventive services in line with “contexts” in patient-centered clinical methods [35]. The study investigated the presence or absence of “family physician’s direct recommendations,” and did not explore doctor-patient communications which could influence the patient’s decisions to get vaccinated. Since the lack of interpersonal communication skills between physicians and their patients was shown to be the primary obstacle in providing preventive medicine [36], further research in this area will be needed.

The study has some limitations. First, due to the nature of a cross-sectional study, a causal relationship between physician’s advice and PPSV23 vaccination cannot be fully understood. Second, there is a possibility that some answer content in the questionnaire might lack accuracy. However, we did conduct the analysis after excluding the data with discordance in vaccination status by reviewing EMR. Third, the relationship between physician’s direct advice and PPSV23 vaccination intention/behavior may be over- or underestimated due to a potential effect of clustering of the physician [37] because a history of interacting with the same physician, or the patient-physician correspondence in the practice, for the participants was not evaluated in the study. In addition, the participants in the study were not a representative sample of Japanese primary care practices and therefore generalizing the inference of the result is limited. Lastly, recall bias could lead to overestimation of the association between physician’s direct advice and PPSV23 vaccination intention/behavior.

After we carried out the study, routine PPSV23 vaccination for the elderly over the age of 65 began in October, 2014 and each municipality started a public expenditure subsidy policy for vaccination. Although there have been no nationwide epidemiological studies to examine the changes in the rates of PPSV23 vaccination as the routine PPSV23 vaccination program was introduced, a piece of research conducted at a community hospital showed introduction of the public expenditure subsidy policy increased by 3.4 times the PPSV23 vaccination rates [38]. Under the current circumstances where availability of a public expenditure subsidy for the vaccination and the country’s support of the routine PPSV23 vaccination program could have increased the PPSV23 vaccination rate, it is unknown to what extent the physician’s advice influences the vaccination behavior.

Currently, we have seen many reports that the provisions of preventive care have been improved by incorporating multidisciplinary cooperation and system-based thinking from those that relied solely on individual recollection and skills. Examples include the introduction of an office system approach based on the characteristics of each medical facility [29], quality improvement activities in clinics, multidisciplinary groups participating in the decision process of a clinic management policy [39], introduction of the reminder system [40], and the presence of the registry [39]. However, these strategies are not necessarily used in medical facilities in Japan, and therefore have a potential to further improve the provisions of preventive medicine in Japanese primary care.

## Conclusion

In a busy family medicine outpatient practice, the family physician’s direct advice was positively correlated with PPSV23 vaccination intention/behavior in the elderly. In order to improve the PPSV23 vaccination rate at the individual level, it is essential to provide information on PPSV23 with better understanding of patient’s perception of the vaccine, using any illness visit as an opportunity for preventive medicine.

## Additional file

Additional file 1: Paper-based Questionnaire. The questionnaire was paper-based and comprised of a total of 22 items, including Health Belief Model related questions, socio-demographic information and multiple choices of reasons for reluctance to get vaccinated. (DOCX 18 kb)

## Abbreviations

EMR: Electronic medical record
HBM: Health Belief Model
MMSE: Mini-Mental State Examination
PCV13: 13-valent pneumococcal conjugate vaccine
PPSV23: 23-valent Pneumococcal Polysaccharide Vaccine
