# Supplementary material for: Correlation between family physician’s direct advice and pneumococcal vaccination intention and behavior among the elderly in Japan: a cross-sectional study
Source: BMC Fam Pract. 2018 Sep 5;19:153. doi: 10.1186/s12875-018-0841-3 (PMC6123972; doi:10.1186/s12875-018-0841-3)
Supplement: Supplementary file 1 — Paper-based Questionnaire. The questionnaire was paper-based and comprised of a total of 22 items, including Health Belief Model related questions, socio-demographic information and multiple choices of reasons for reluctance to get vaccinated. (DOCX 18 kb) [file 12875_2018_841_MOESM1_ESM.docx]

**Question 1**

Do you know what pneumococcal vaccine is?

1. Yes, I do.

2. No, I don’t.

**Question 2**

Have you ever been recommended to get a pneumococcal vaccine by your doctor when you came to our clinic?

1. Yes, I have.

2. No, I haven’t.

3. I don’t know.

**Question 3**

Have you ever gotten a pneumococcal vaccine?

1. Yes, I have.

2. No, I haven’t.

3. I don’t remember.

(Only for those who answered “No” in question 3.)

**Question 4**

What do you currently think about getting a pneumococcal vaccine?

1. I plan to get vaccinated in the future.

2. I have decided not to get vaccinated.

3. I don’t know if I will get vaccinated or not.

(Only for those who chose 2 and 3 in question 4.)

**Question 5**

What are your reasons for not getting a pneumococcal vaccine? Please circle all the numbers that apply.

1. I wasn’t recommended to get a pneumococcal vaccine by my doctor.

2. I didn’t clearly understand the effects of the vaccine.

3. I didn’t have time to get vaccinated.

4. I don’t think I need to get vaccinated because I am healthy.

5. I don’t think it’s effective.

6. Due to the cost of the vaccine.

7. I’m worried about the side effects of the vaccine.

8. I don’t know where I can go to get vaccinated. / There are no locations nearby where I can get vaccinated.

9. I’m afraid of / hate injections.

10. I’m not interested in getting vaccinated.

11. I wasn’t feeling well.

12. Other reasons (please specify). ( )

**Question 6**

Do you know about the public vaccination subsidy provided by your municipality to help you cover the cost of the pneumococcal vaccine?

1. Yes, I do.

2. No, I don’t.

**Question 7**

Do you think you tend to catch colds easily?

1. Absolutely yes.

2. To a certain extent.

3. Moderate.

4. Not really.

5. I don’t think so.

**Question 8**

Do you think you are susceptible to getting pneumonia?

1. Absolutely yes.

2. To a certain extent.

3. Moderate.

4. Not really.

5. I don’t think so.

**Question 9**

If you got pneumonia, do you think you would develop a severe case?

1. Absolutely yes.

2. To a certain extent.

3. Moderate.

4. Not really.

5. I don’t think so.

**Question 10**

Have you ever had pneumonia?

1. Yes.

2. No.

3. I don’t know.

**Question 11**

Do you suffer/have you suffered from any lung (respiratory) diseases, aside from pneumonia, for which you have received medical treatments or require regular visits to your doctor?

1. Yes. (Asthma, emphysema, chronic obstructive pulmonary disease (COPD), tuberculosis, etc.)

2. No.

**Question 12**

Have any of your family members gotten pneumonia before?

1. Yes.

2. No.

3 I don’t know.

**Question 13**

Do you think pneumococcal vaccines are effective?

1. Absolutely yes.

2. To a certain extent.

3. Moderate.

4. Not really.

5. I don’t think so.

**Question 14**

Do you think the cost of pneumococcal vaccine would be a financial burden to you? If you have already been vaccinated before, did you feel it was too expensive?

1. Absolutely yes.

2. To a certain extent.

3. Moderate.

4. Not really.

5. I don’t think so.

**Question 15**

Do you live alone?

1. Yes, I do.

2. No, I don’t.

**Question 16**

Do you need your family’s assistance when you come to our clinic and when you go home?

1. Yes, I do.

2. No, I don’t.

**Question 17**

How often do you go to a doctor for medical check-ups?

1. Once a year or every other year.

2. Once every 3 to 4 years.

3. I went over 5 years ago but haven’t been since.

4. I have never done it before.

**Question 18**

Do you currently smoke?

1. Yes, I do.

2. No, I don’t.

**Question 19**

How would you rate your current health condition?

1. Good.

2. Somewhat good.

3. Average.

4. Not so good.

5. Not good.

**Question 20**

What was your highest level of education?

1. Elementary school or junior high school.

2. High school.

3. Vocational school or junior college.

4.University or graduate school.

**Question 21**

How would you describe your family’s standard of living?

1. Very comfortable.

2. Moderately comfortable.

3. Average.

4. Somewhat struggling.

5. Low.

**Question 22**

Did anyone help you with reading or answering this questionnaire?

1. I had someone help me. (for reading and/or answering this questionnaire.)

2. I answered the questions myself.
